# Polypyrimidine Tract Binding Protein-1 (PTB1) Is a Determinant of the Tissue and Host Tropism of a Human Rhinovirus/Poliovirus Chimera PV1(RIPO)

**DOI:** 10.1371/journal.pone.0060791

**Published:** 2013-04-04

**Authors:** Nusrat Jahan, Eckard Wimmer, Steffen Mueller

**Affiliations:** Department of Molecular Genetics and Microbiology, Stony Brook University, Stony Brook, New York, New York, United States of America; University of Hong Kong, Hong Kong

## Abstract

The internal ribosomal entry site (IRES) of picornavirus genomes serves as the nucleation site of a highly structured ribonucleoprotein complex essential to the binding of the 40S ribosomal subunit and initiation of viral protein translation. The transition from naked RNA to a functional "IRESome" complex are poorly understood, involving the folding of secondary and tertiary RNA structure, facilitated by a tightly concerted binding of various host cell proteins that are commonly referred to as IRES trans-acting factors (ITAFs). Here we have investigated the influence of one ITAF, the polypyrimidine tract-binding protein 1 (PTB1), on the tropism of PV1(RIPO), a chimeric poliovirus in which translation of the poliovirus polyprotein is under the control of a human rhinovirus type 2 (HRV2) IRES element. We show that PV1(RIPO)'s growth defect in restrictive mouse cells is partly due to the inability of its IRES to interact with endogenous murine PTB. Over-expression of human PTB1 stimulated the HRV2 IRES-mediated translation, resulting in increased growth of PV1(RIPO) in murine cells and human neuronal SK-N-MC cells. Mutations within the PV1(RIPO) IRES, selected to grow in restrictive mouse cells, eliminated the human PTB1 supplementation requirement, by restoring the ability of the IRES to interact with endogenous murine PTB. In combination with our previous findings these results give a compelling insight into the thermodynamic behavior of IRES structures. We have uncovered three distinct thermodynamic aspects of IRES formation which may independently contribute to overcome the observed PV1(RIPO) IRES block by lowering the free energy δG of the IRESome formation, and stabilizing the correct and functional structure: 1) lowering the growth temperature, 2) modifying the complement of ITAFs in restricted cells, or 3) selection of adaptive mutations. All three mechanisms can conceivably modulate the thermodynamics of RNA folding, and thus facilitate and stabilize the functional IRES structure.

## Introduction

**Figure 1:**
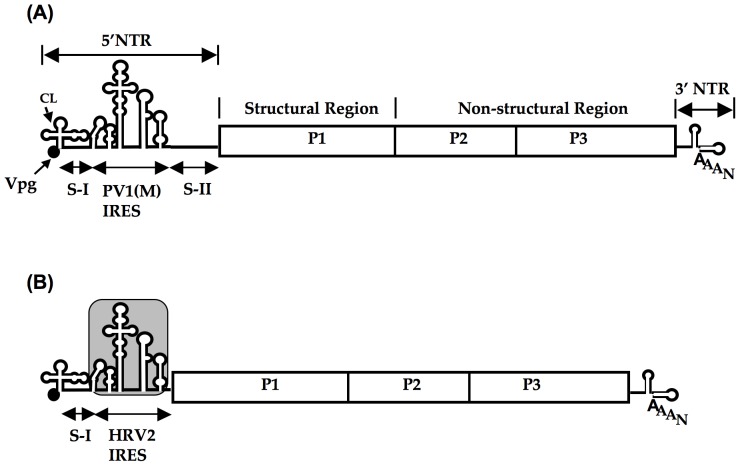
Structure of PV1(M) and PV1(RIPO) genome. (A) Schematic representation of PV1(M) genome. CL: Cloverleaf, 5′ NTR: 5′ non-translated region, 3′ NTR: 3′ non-translated region, IRES: internal ribosomal entry site, S-I: spacer I, and S-II: spacer II. (B) Schematic representation of PV1(RIPO) genome. PV1(RIPO) has the IRES of HRV2 (shaded box) instead of the IRES of PV1(M).

Apart from ribosomal RNAs, internal ribosomal entry sites (IRESs) of many plus strand RNA viruses are the most intriguing RNA structures in biological systems [1], [2], [3], [4], [5], [6], [7]. IRESes of plus strand viruses mapping to the 5′ non-translated region of the sequence (Fig. 1A) are defined by function, not by structure [8]. Their function is to promote initiation of cap-independent initiation of translation immediately after the viral genome has entered the cell. Cellular IRESes that function in regulating cellular protein synthesis have also been identified but a structural relationship between viral and cellular IRESes has not been established.

Just like ribosomal RNAs, the genomes forming an IRES are biologically active only when complexed to specific sets of proteins, referred to as IRES *trans*-acting factors (ITAFs) [9]. ITAFs required for viral IRES function are, surprisingly, of cellular and not of viral origin. This makes sense, however, since an IRES of a plus strand RNA virus must function prior to virus-specific protein synthesis [1], [3]. This notion is supported also by the findings from Dobrikova *et al.* [10], where the authors showed that viral IRES function in a properly configured template in uninfected cells although IRES efficiency benefit from expression of viral gene products [10]. The high error rate in RNA replication has dictated that viral RNA genomes are small [11] yet, remarkably, their IRES elements are relatively large. Together with ITAFs, viral IRESes form structures, referred to as IRESomes [12], [13]. Since ITAFs may be in short supply or absent in specific cell types, IRESomes may confer tissue tropism to a virus [14], [15], [16], [17], [18], [9], [19], [20]. The most commonly found ITAFs in IRESomes of picornaviruses are polypyrimidine tract-binding protein (PTB) [21], [22], [23], [24], [25], [26], [27], [28]; poly(rC)-binding protein 2 (PCBP2) [29], [30]; the autoantigen La [31], [32], [33], [34], [35]; the upstream of N-ras protein (unr) [24], [25], [36]; and ITAF45 [9].

IRES elements can be exchanged between picornaviruses in spite of their structural differences which results in the formation of different chimeric viruses [17], [20], [37], [38], [39], [40], [41], [42]. Propagated on HeLa cells, these chimeras usually don’t exhibit any replication phenotypes. This, however, may not be true when the chimera is propagated in specific tissue culture cells. In contrast to the parental poliovirus type 1 (Mahoney) [PV1(M)], the chimeric virus PV1(RIPO) harboring the IRES of HRV2 instead of the IRES of PV1(M) (Fig. 1B), shows a distinct tissue and host tropism when grown in human cells of neuronal origin [17], [40], [41], [43], [44], or, most dramatically, in mouse cells [20]. Restriction of growth of PV1(RIPO) in mouse cells, however, is observed only at physiological temperature; at 33°C PV1(RIPO) grows with wild type (wt) kinetics [20]. This peculiar temperature sensitive (ts) phenotype could be due to either the qualitative or the quantitative differences of the ITAFs present in cells of mouse origin, for example, an inability of the mouse-specific ITAFs to function in the context of the HRV2 IRES at physiological temperature. A neuronal cell-specific homologue of PTB (nPTB) in the mouse CNS has been described to function in the neurovirulent phenotype of Theiler's murine encephalitis virus (TMEV) [19], [45], [46], [47], [48], a cardiovirus of *Picornaviridae*. Moreover, a mouse homologue of PTB (mPTB) [48], [49], [50] has been implicated in the regulation of early mouse development through IRES dependent translation of a protein in mouse embryonic stem cells [51], [52].

Three alternatively spliced isoforms of human PTB (hPTB) has been described, PTB1, PTB2, and PTB4 [53], [23]. Although hPTB is required for poliovirus and rhinovirus IRES function, it is not known how mPTB would interact with the IRESes of these viruses. The HRV2 IRES of PV1(RIPO) might assume a conformation at 37°C in mouse cells that is insufficient for initiation of translation due to a different menu of ITAFs. In this study we have analysed the possibility of whether PV1(RIPO) replication in mouse cells at physiological temperature can be rescued by production of human PTB 1 (hPTB1).

## Materials and Methods

### Viruses and cells

The neurovirulent PV type 1 [Mahoney; PV1(M)] is a strain being used routinely in the laboratory [54]. PV1(RIPO) was constructed as described previously [17]. Briefly, PV1(RIPO) is a poliovirus in which it's IRES has been replaced by that of rhinovirus type 2 (HRV2) while spacer II of PV1 was deleted (Fig. 1). R-1235 virus is mouse L20B cell-adapted PV1(RIPO) which was obtained after seven serial passages at 37°C [20]. The mouse fibroblast cell lines (L cells) expressing CD155δ (L20B) [55], [56], were maintained in Dulbecco's Modified Eagle medium (DMEM) containing 1% penicillin/streptomycin and 10% bovine calf serum (BCS). Human neuroblastoma cells SK-N-MC, cells of neurogenic origin, were obtained from the American Type Culture Collection (Manassas, VA) and were maintained according to the supplier's specification. HeLa R19 cells, a strain of human cervical carcinoma cells [57] that readily forms monolayers, is routinely used in the laboratory [58].

### Expression of hPTB1

SK-N-MC cells and L20B cells were transfected using Lipofectamine 2000 (Invitrogen) with the mammalian expression plasmid pCDNA3. This plasmid contained the gene for hPTB1 (polypyrimidine track binding protein 1 [22]) expressed under the control of cytomegalovirus promoter. The transfection procedure was followed as outlined by the manufacturer. Stable cell clones resistant to G418 were selected.

### Antibodies

Anti-PTB monoclonal antibodies DH1, DH3, DH7 and DH17 [59], and anti-actin mouse mAb (Calbiochem) were used as primary antibodies for Western blot analysis.

### Immunoblot analysis

For immunoblot analysis, an aliquot of the total cell extract containing 30 µg of protein was separated by sodium dodecyl sulfate (SDS) gel (12% acrylamide) electrophoresis. Following electrotransfer to polyvinylidene difluoride membranes, membranes were blocked with 5% skim milk in 0.1% Tween in phosphate buffered saline (PBS) for 0.5 h. Membranes were probed with anti-PTB monoclonal antibodies at a 1∶200 dilution overnight at 4°C. The membranes were washed three times and incubated with horseradish peroxidase-conjugated anti-mouse immunoglobin G for 2 h. After three washes, proteins were visualized with an enhanced chemiluminescence reagent kit (Amersham International Plc.) according to the manufacturer's recommended procedure. The blot was stripped and re-used in order to detect actin using anti-actin (Ab-1) mouse mAb (JLA20) (Calbiochem) as the primary antibody and goat anti-mouse IgM conjugated to horseradish peroxidase (Calbiochem) as the secondary antibody.

### Growth of virus at 37°C and 39.5°C

Cell monolayers in 35 mm plastic culture dishes were washed with DMEM and inoculated at an MOI of 10 (10 PFU/cell) with the virus to be tested. The dishes were rocked for 30 min at room temperature, the cells were thoroughly washed to remove unbound virus and placed at 37°C and 39.5°C. At 0 and 24 hr post infection (p.i.), the dishes were subjected to three consecutive freeze-thaw cycles, and the viral titers of the supernatants were determined by plaque assay, as previously described [58].

### Poliovirus luciferase replicons and luciferase assay

A wt replicon PV1(M)-luc, in which the PV capsid coding sequence P1 was replaced by the firefly luciferase gene was previously constructed [60]. An analogous chimeric replicon PV1(RIPO)-luc was made, which carries the wt HRV2 IRES instead of the PV IRES [20]. The plasmids containing these replicons were linearized with *Dra*I and used for RNA synthesis using phage T7 RNA polymerase. *In vitro* transcribed replicon RNA was transfected into monolayers (35-mm-diameter dishes) of SK-N-MC, SK-N-MC^hPTB1^, L20B, and L20B^hPTB1^ cells; using a modified DEAE-Dextran transfection method [61] and incubated at 37°C in DMEM, 2% BCS. At different times points post-transfection, the growth medium was removed from the dishes and the cells were washed gently with 2 ml of PBS. The cells were lysed and the firefly luciferase activity was measured by methods described previously [62].

### Fluorescence microscopic analysis

L20B and L20B^hPTB1^ cells were seeded and co-cultured on 20-mm coverslips to 80% confluency and were infected with PV1(RIPO), PV1(M), and R-1235 at MOI of 10. At six hours post infection, cells were washed with PBS and fixed with 4% paraformaldehyde for 30 min at room temperature. Cells were then washed with PBS and permeabilized using 0.4% Triton X-100 for 5 min at room temperature. After another PBS wash, cells were incubated in blocking solution (10% BSA in PBS) for 30 min at 37°C and immunostained with anti-PTB (mouse monoclonal), anti-PV1 (rabbit polyclonal) for 1 hour at 37°C. After they had been washed six times with PBS, cells were incubated with Cy3-conjugated goat anti-mouse IgG (Invitrogen) and Alexa488-conjugated goat anti-rabbit IgG for 1 h at 37°C. The cells were then observed under a fluorescent microscope.

### Preparation of cytoplasmic extracts

L20B and L20B^hPTB1^ cells were grown in DMEM containing 10% (v/v) BCS and 1% penicillin/streptomycin. Whole cells were grown to 90% confluence and washed three times with PBS. The cytoplasmic extracts of cells were then prepared as previously described [58].

### RNA binding assay

To test the binding of PTB to PV RNA fragments derived from the 5′NTR of PV1(RIPO), PV1(M), and R-1235 (Clover-leaf to stem-loop domain VI), RNA pull-down assays were used similarly to those described by Toyoda *et al.* [63]. To generate biotinylated RNA probes, one µg of DNA fragments was used to prepare RNA transcripts using T7 RNA polymerase (Stratagene) with nucleoside triphosphates in biotinylation buffer (1 mM ATP, 1 mM CTP, 1 mM GTP, 0.65 mM UTP, and 0.35 mM biotin-16-UTP [Roche]). After 2 h incubation at 37°C, 5 U of RNase-free DNase (Roche) was added in order to remove the template DNA. The transcripts RNAs were purified by phenol-chloroform extraction and ethanol precipitation. RNA pull-down experiments were performed with cell extracts containing 800 µg of protein and 4 µg of biotinylated RNAs. After incubation of the RNA-protein mixture in 1 ml of incubation buffer (10 mM HEPES [pH 7.4], 1.5 mM magnesium acetate, 90 mM potassium acetate, 2.5 mM dithiothreitol, 0.05% NP-40) for 30 min at 4°C, the samples were subjected to streptavidin-agarose resin (Pierce) adsorption and incubated for an additional 2 h. As a nonspecific competitor 20 µg of yeast tRNA (Roche) was added to the binding mixtures. After incubation, the resin was washed four times with incubation buffer, and then the resin-bound proteins were resolved by SDS-12.5% polyacrylamide gel electrophoresis. Western blot analysis was performed with anti-PTB monoclonal antibodies.

## Results

In contrast to its replication in HeLa R19 cells, PV1(RIPO) proliferation was shown to be inhibited in human neuroblastoma cells (SK-N-MC) at 37°C [17]. Furthermore, PV1(RIPO) had lost the neurovirulent phenotype of PV1(M) in mice transgenic for the human PV receptor gene, *CD155* (CD155tg mice), and in non-human primates [17], [40]. The relationship between genotype and phenotype(s) became even more complex when it was found that the R-1 variant of PV1(RIPO), in which a restriction site sequence within spacer I of the poliovirus 5′ NTR was deleted, grew well in SK-N-MC cells yet showed high neuroattenuation in *CD155* tg mice [20]. Therefore, we concluded that replication in SK-N-MC cells and neurovirulence in *CD155* tg mice do not necessarily co-vary. We also reported that PV1(RIPO) possesses a strong temperature dependent growth defect in all CD155 transgenic mouse cell lines tested (both neuronal and non-neuronal) [20]. If the observed tissue and host tropism is related to the quality or quantity of on or more ITAFs, over-expression of an ITAF may result in rescue of IRES function of PV1(RIPO) even in neuroblastoma cells or in mouse cells at 37°C.

### Over-expression of human polypyrimidine tract binding protein-1 (hPTB1) in SK-N-MC cells stimulates proliferation of PV1(RIPO)

**Figure 2:**
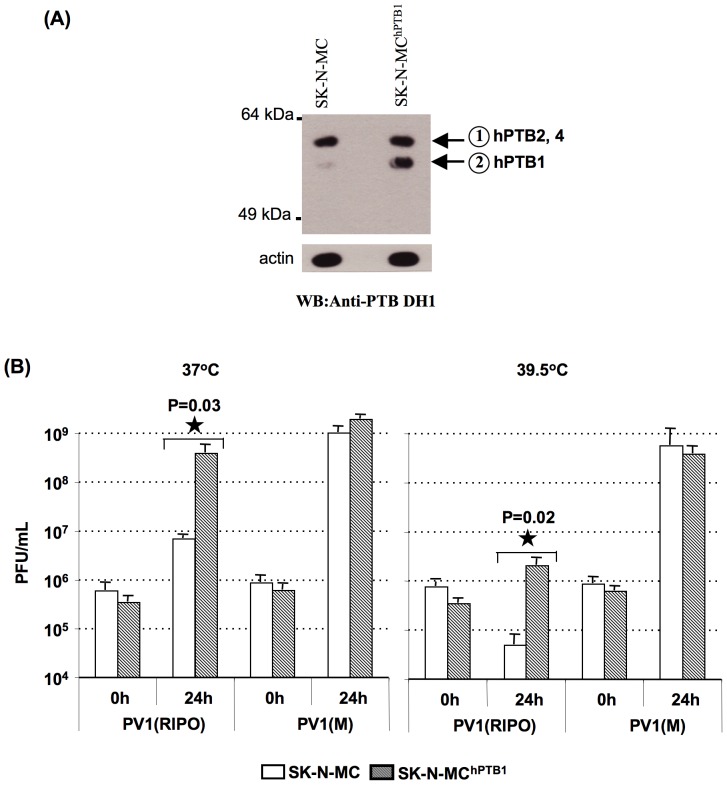
Over-expression of hPTB1 in SK-N-MC cells and its effect on viral growth. (A) Expression of different isoforms of human PTB (hPTB) isoforms in the parental SK-N-MC and SK-N-MC^hPTB1^ cells was shown using monoclonal antibodies to PTB. hPTB2 and hPTB4 with a molecular weight of ∼58 kDa are indicated by arrow numbered 1 and hPTB1 with an apparent molecular mass of ∼57 kDa is indicated by arrow numbered 2. Actin was detected as a loading control. (B) Comparison of the growth phenotype of PV1(RIPO) and PV1(M) in the parental SK-N-MC cells and with that in SK-N-MC^hPTB1^ cells at 37°C and 39.5°C, at an MOI of 10. The bars show the average and the standard deviation of three independent experiments. In all cases, averages±SD of at least three independent experiments are shown; <$>\raster="rg1"<$> denotes student t-test p value <0.05.

To determine whether a quantitative difference in hPTB1 would stimulate PV1(RIPO) growth in human neuronal cells, derivatives of SK-N-MC cells stably over-expressing hPTB1 were generated (SK-N-MC^hPTB1^) (see Materials and Methods). Immunoblot assays were carried out to assess the level of expression of hPTB1 in these cells, as compared to their parental SK-N-MC cells (Fig. 2A). In the parental SK-N-MC cells, PTB was seen as a double band migrating with an apparent molecular mass of ∼57 kDa (indicated by arrows numbered 1 and 2 in Fig. 2A). The band indicated by arrow numbered 2 in Fig. 2A, representing hPTB1, was significantly intensified in the SK-N-MC^hPTB1^ cells.

The extent to which the over-expression of hPTB1 may rescue the growth defect of PV1(RIPO) was examined by growing PV1(RIPO) in the over-expressing cells and comparing its growth phenotype in these cells with that in the restrictive parental cells. As seen in Fig. 2B, the growth of PV1(RIPO) is significantly enhanced in SK-N-MC^hPTB1^ cells at 37°C. Furthermore, the restricted growth phenotype of PV1(RIPO) is partially rescued in these cells at 39.5°C. Interestingly, the growth of PV1(M) was unaffected by the over-expression of hPTB1. This result suggests that the amount of PTB or the other ITAFs in SK-N-MC cells are sufficient for the optimal growth of PV1(M) in these cells.

### Expression of hPTB1 rescues the growth defect of PV1(RIPO) in mouse L20B cells

**Figure 3:**
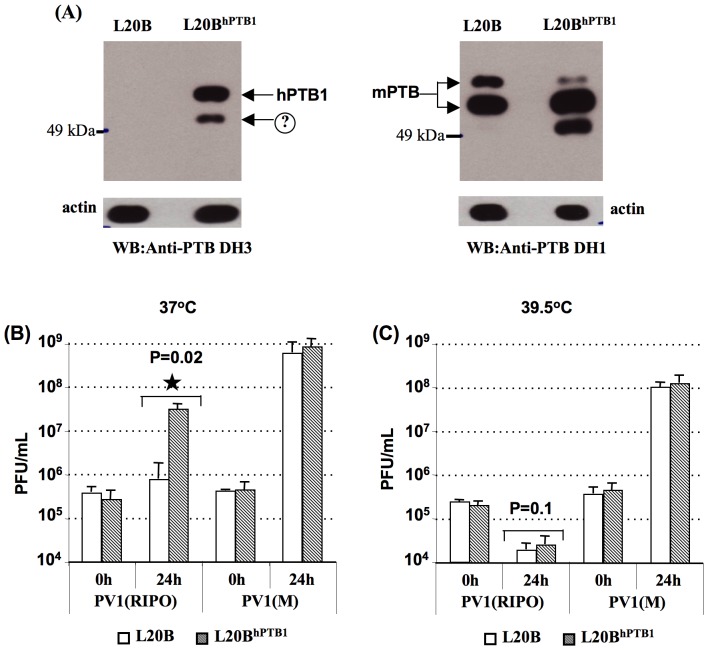
Expression of hPTB1 in L20B cells and its effect on viral growth. (A) Expression of PTB in parental L20B cells versus L20B^hPTB1^ cells was shown using two different monoclonal antibodies to PTB. Left blot: The upper band migrating with an apparent molecular mass of ∼57 kDa represents the exogenously expressed hPTB1. A new minor PTB species migrating with a slightly smaller apparent mass than the major band is indicated with a question mark (?). Right blot: The antibodies used here detected the endogenous mouse PTB (mPTB) in addition to the exogenous hPTB1. Actin was detected as a loading control. (B) and (C) Comparison of the growth phenotype of PV1(RIPO) and PV1(M) in the parental L20B cells and with that in L20B^hPTB1^ cells at 37°C and 39.5°C, respectively, at an MOI of 10. In all cases, averages ± SD of at least three independent experiments are shown; <$>\raster="rg1"<$> denotes student t-test p value <0.05.

PV1(RIPO) has a severe propagation defect in mouse cells [20]. Therefore, we were interested in examining the effect of hPTB1 on PV1(RIPO) growth in L20B cells, a mouse fibroblast cell line expressing the PV receptor, CD155. The expression of hPTB1 in L20B cells stably expressing hPTB1 (L20B^hPTB1^) was determined by immunoblot assays using monoclonal antibodies to hPTB1 and was compared with PTB expression in parental L20B cells (Fig. 3A). Among four different monoclonal antibodies to hPTB1, anti-PTB DH1 (Fig. 3A, right blot), DH7 and DH17 (not shown) were able to detect both the recombinant hPTB1 (expressed in L20B^hPTB1^cells) and endogenous mouse PTB (mPTB, in L20B cells) (Fig. 3A, right blot). Interestingly, a fourth monoclonal antibody, anti-PTB DH3, only detected the recombinant hPTB1 expressed in L20B cells, but not the endogenous mPTB isoforms (Fig. 3A, left blot). Thus, it can be inferred that mAb DH3 is specific for the hPTB1, which represents the antigen used for production of this series of anti PTB mAbs [59]. A new minor PTB species migrating with a slightly smaller apparent mass than the major bands (indicated by arrow with a question mark in Fig. 3A) was observed. It is likely that this variant form is derived from the transfected hPTB1, and represents either a post-translational modification that results in altered gel mobility (such as a phosphorylation event, or a cleavage product of hPTB1.).

To determine whether the production of hPTB1 in L20B cells plays a role in the growth of PV1(RIPO), the growth of this virus was compared with that of PV1(M) in L20B cells and L20B^hPTB1^ cells (Fig. 3B, 3C). Expression of hPTB1 protein in L20B cells rescued the defective growth of PV1(RIPO) at 37°C (Fig. 3B) but no rescue was observed at 39.5°C (Fig. 3C). The expression of hPTB1 failed to show any influence on PV1(M) growth (Fig. 3B and Fig. 3C). This observation suggests that the PV IRES is either capable of using endogenous mPTB or, less likely, is not dependent of PTB in mouse cells.

### Mouse L20B cell-adapted PV1(RIPO) is non-responsive to hPTB1 supplementation

**Figure 4:**
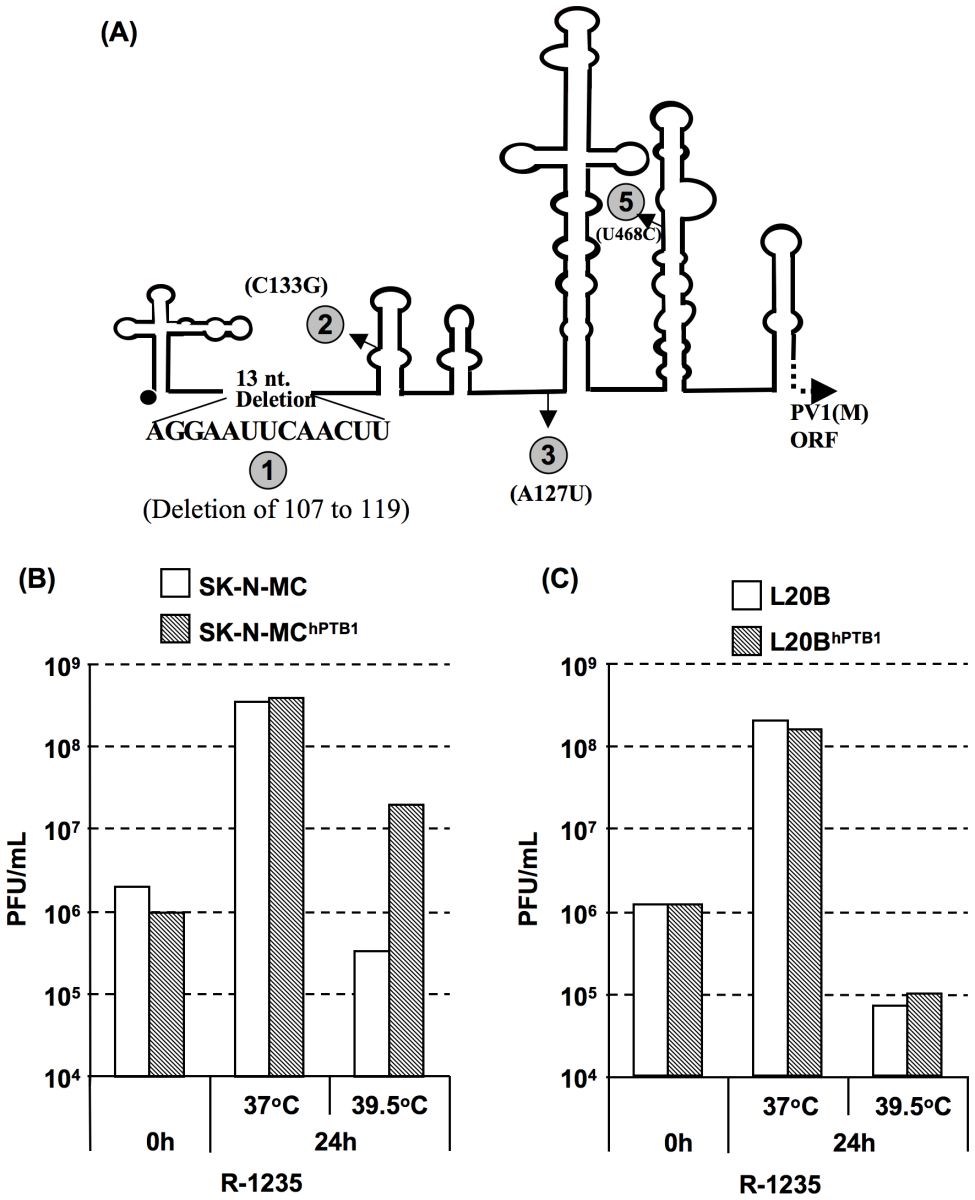
Effect of hPTB1 supplementation on the growth of R-1235 virus. (A) Schematic representation of the IRES of R-1235 virus. The changes in nucleotides in the 5′NTR and in the IRES are indicated and they are numbered as 1, 2, 3, and 5. (B) Comparison of the growth phenotype of R-1235 virus in the parental SK-N-MC cells and with that in SK-N-MC^hPTB1^ cells at 37°C and 39.5°C, at an MOI of 10. The bars show the average of two independent experiments. (C) Comparison of the growth phenotype of R-1235 virus in the parental L20B cells and with that in L20B^hPTB1^ cells at 37°C and 39.5°C, at an MOI of 10. The bars show the average of two independent experiments.

A variant of PV1(RIPO) adapted to grow in mouse cells, described previously, showed high neurovirulence in *CD155* *tg* mice [20]. This variant, R-1235, contains four mutations in the 5′ NTR (Fig. 4A). R-1235 showed wt PV1(M)-like growth in SK-N-MC cells and L20B cells, therefore it was interesting to examine the effect of hPTB1 supplementation on the growth of this virus in these cells. As was observed with wt PV1(M), expression of hPTB1 in SK-N-MC cells or L20B cells had also no influence on the growth of R-1235 in these cells at 37°C (Fig. 4B, 4C). In contrast, hPTB1 expression caused a 10-fold increase in R-1235 titer in SK-N-MC cells at 39.5°C (Fig. 4B) whereas no increase in L20B cells at 39.5°C (Fig. 4C). We conclude that R-1235 has either become adapted to the use of endogenous mPTB or lost its dependence on PTB in L20B cells only at 37°C, the temperature at which this variant was selected.

### Rescue of HRV2 IRES-mediated translation defect by expression of hPTB1 in L20B cells

**Figure 5:**
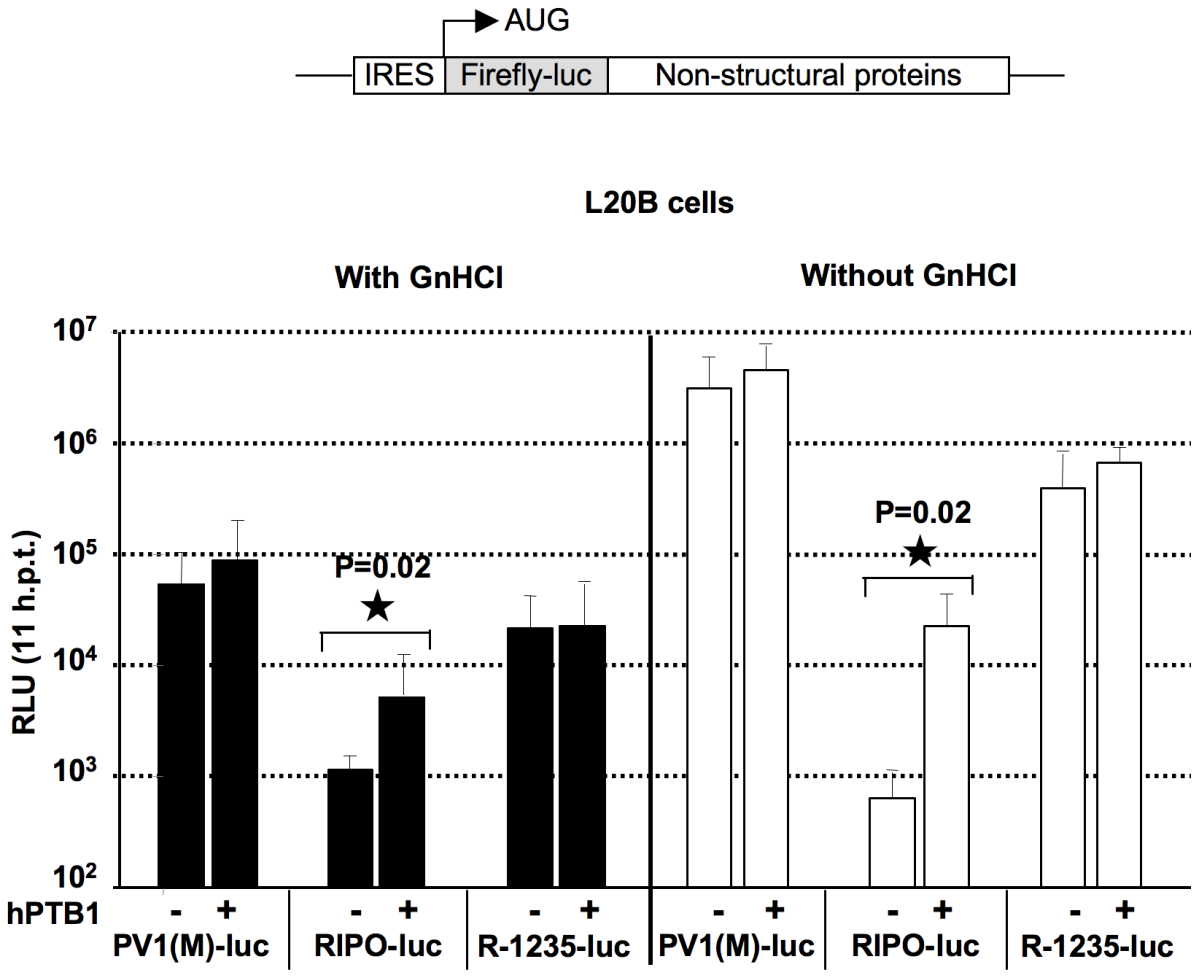
RNA translation and replication of PV1(M)-luc, PV1(RIPO)-luc, and R-1235-luc replicons. Monolayers of mouse L20B and L20B^hPTB1^ cells were transfected with *in vitro* transcribed RNA of luciferase replicons and incubated at 37°C in the presence (for translation) or in the absence (for replication) of 2mM guanidine hydrochloride (GnHCl). RNA translation and RNA replication were assessed by measuring the luciferase activity at 11 h post transfection. In all cases, averages ± SD of at least three independent experiments are shown; <$>\raster="rg1"<$> denotes student t-test p value <0.05.

The differences in the interaction of mPTB and hPTB1 with HRV2 IRES might play an important role in the HRV2 IRES-mediated translation and thus the outcome of PV1(RIPO) infection in mouse L20B cells or in the experimental mouse model. Therefore, we sought to determine the extent to which hPTB1 expression mediates enhancement of translation directed by the HRV2 IRES in L20B cells. For this purpose, the functionality of subgenomic PV replicons, specifically, PV1(M)-Luc and PV1(RIPO)-Luc, expressing the firefly luciferase reporter gene in place of the PV capsid proteins, was analyzed and reported previously [20]. To differentiate between the luciferase signals due to translation from the incoming viral RNA and signals due to translation from mRNA synthesized during replication, the cells were grown in the presence and absence of 2 mM guanidine hydrochloride (GnHCl). At this concentration, GnHCl inhibits viral RNA replication without any toxic effect on cellular processes or viral translation [64], [65], [66]. HRV2 IRES-mediated translation and RNA replication were measured in regular L20B cells and L20B^hPTB1^ cells by transfecting the cells with *in vitro* transcribed RNA of PV1(M)-Luc and PV1(RIPO)-Luc PV luciferase replicons and incubating the cells at 37°C in the presence (for translation) or in the absence (for replication) of 2 mM GnHCl (Fig. 5). A 10-fold enhancement of HRV2-directed translational activity was obtained in L20B^hPTB1^ cells (Fig. 5) causing more than a 100-fold increase in replication of PV1(RIPO)-luc replicon in L20B^hPTB1^ cells. This increase in HRV2 IRES-mediated translation and RNA replication of PV1(RIPO)-luc replicon in L20B^hPTB1^ cells is significant. These results are consistent with the effect of hPTB1 on PV1(RIPO) virus growth in L20B^hPTB1^ cells (Fig. 3B). The increase in HRV2 IRES-directed translation in the presence of hPTB1 in L20B cells again suggests that the mPTB present in L20B cells is insufficient for optimal function of the HRV2 IRES. These results also indicate that the expression of hPTB1 compensates for the intrinsically poor activity of mPTB on HRV2 IRES activity in L20B cells.

Consistent with the growth of the PV1(M) (Fig. 3B) and R-1235 (Fig. 4C), the IRES activity of PV1(M) and R-1235 is non-responsive to the absence or presence of hPTB1 in L20B or L20B-PTB cells, respectively (Fig. 5). We conclude that the PV IRES is either capable of using mouse PTB or, less likely, is independent of PTB in mouse cells.

### Mouse cell-adapted PV1(RIPO) no longer requires hPTB supplementation

**Figure 6:**
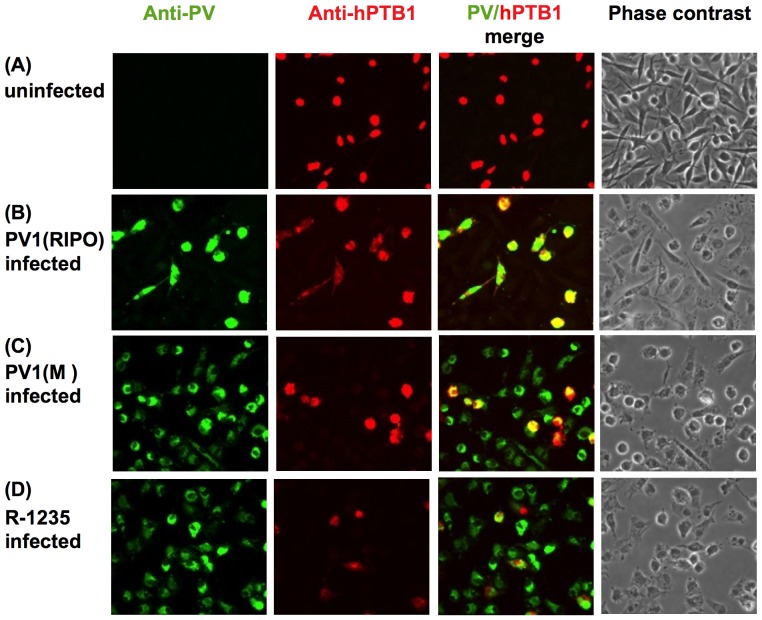
Infection on L20B/L20B^hPTB1^ co-cultured cells with PV1(M), PV1(RIPO) and R-1235 virus. (A) Uninfected, (B) PV1(RIPO) infected, (C) PV1(M) infected, and (D) R-1235 infected co-culture of L20B/L20B^hPTB1^ cells were immunostained with antibodies against PV capsid proteins and hPTB1 at 6 hours post infection.

There are several possible explanations of the preceding observations: (i) PV1(RIPO) is rescued by hPTB1 in L20B^hPTB1^ cells because it cannot use mPTB in L20B cells, (ii) PV1(M) and R-1235 can use mPTB in L20B cells, or (iii) PV1(M) and R-1235 may be independent of PTB in L20B cells. To assess the validity of these possibilities, we analyzed the growth of wild type PV1(M), PV1(RIPO) and R-1235 in a co-culture of L20B and L20B^hPTB1^. We reasoned that it might be possible to detect a clear evidence of a population of L20B cells expressing hPTB1 in a co-culture in the background of hPTB1-negative L20B cells. The co-culture was infected with PV1(M), PV1(RIPO), or R-1235 for 6 hrs at 37°C and the infected cells were examined for the expression of PV antigens and status of hPTB1 expression (Fig. 6). In the uninfected co-culture of L20B/L20B^hPTB1^ cells hPTB1 expression was observed only in the L20B^hPTB1^ cells due to use of an hPTB-specific antibody (Fig. 6A). Despite a multiplicity of infection of 10 PFU/cell, PV1(RIPO) was able to replicate only in L20B^hPTB1^ (Fig. 6B). Consequently infection of the parental L20B cells leads to undetectable or abortive infection by PV1(RIPO). This result clearly shows that hPTB1 supplementation helped PV1(RIPO) to overcome the growth restriction in mouse cells. In contrast, productive infection was observed with the wt PV1(M) (Fig. 6C) and the adapted PV1(RIPO), R-1235 (Fig. 6D), irrespective of the status of the hPTB1 expression in L20B cells. This indicates that these viruses are either able to use mPTB or are independent of PTB in mouse cell line L20B.

### PV1(RIPO) uses hPTB1 in L20B^hPTB1^ cells and cannot use mPTB whereas PV1(M) and R-1235 are able to use both mPTB and hPTB1

**Figure 7:**
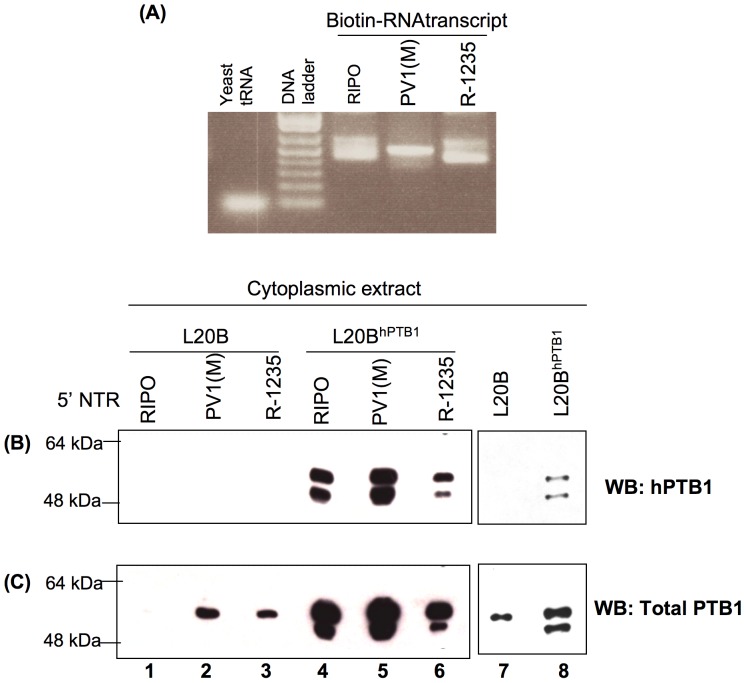
Analysis of PTB binding to 5′NTR RNA fragments of PV1(RIPO), PV1(M), and R-1235. (A) The comparative levels of the 5′NTR RNA probes of PV1(RIPO), PV1(M), and R-1235 used for the pull-down were analysed by agarose gel-electrophoresis. PTB binding to biotinylated 5′NTR RNA probes of PV1(RIPO), PV1(M), and R-1235 was analyzed with an RNA pull-down assay followed by Western analysis with anti-PTB monoclonal antibodies against hPTB1(B) or total PTB (C) (see Materials and Methods). Lanes 1 to 6 represents the PTB pulled down by the corresponding 5′NTR RNA probes. Lanes 7 and 8 represents the amount of PTB present in 2% of the used cell-extracts in the RNA pull-down assay.

To further our understanding of the role of either hPTB1 in L20B^hPTB1^ cells or mPTB in L20B cells, the physical interaction of hPTB1 and mPTB with the PV IRES and HRV2 IRES was compared in parallel by performing an RNA binding assay. The RNA Binding assay, as described in the Materials and Methods, was performed by using biotinylated probes equivalent to the 5′NTR of PV1(M), PV1(RIPO), and R-1235, including their 5′-terminal cloverleaf and IRES (Fig. 7). The comparative levels of the biotinylated probes used for the pull-down is shown in Figure 7A. The result from the binding assay shows that the 5′NTR of PV1(RIPO) is unable to bind endogenous mPTB (Fig. 7C lane 1) but can bind to hPTB1 expressed in L20B^hPTB1^ cells (Fig.7B lane 4). In sharp contrast, the 5′NTR of PV1(M) can bind both hPTB1 (Fig. 7B lane 5) and mPTB (Fig. 7C lane 2). Similarly, R-1235 also is able to bind hPTB1 (Fig. 7B lane 6) and mPTB (Fig. 7C lane 3). Compared to PV1(M), it seems that R-1235 and RIPO interact less efficiently with hPTB1, whereas PV1(RIPO) does not interact at all with mPTB1 This suggests that the major impact of the adaptation mutations could have been to increase IRES stability. These data combined provide compelling evidence that the observed PV1(RIPO) phenotype in mouse cells is partially due to it's inability to interact with mPTB, while wt PV or a mouse-adapted PV1(PIPO) variant, R-1235, are capable of utilizing mouse PTB for the proper formation of the “IRESome”.

## Discussion

IRES-dependent tropism between cell types of different hosts can be determined largely by the quantitative and qualitative differences in some non-canonical RNA binding cellular factors. These factors, commonly known as ITAFs, are believed to stabilize or alter the IRES structure to fold the IRES into the correct conformation and promote the process of ribosome internal binding to the IRES element. The expression of ITAFs in a tissue-specific manner may be responsible for determining the activity of viral IRESes and the growth of the virus in different cell types. Polypyrimidine tract binding protein has been reported to interact at a number of sites within the picornaviral IRESes and to stimulate IRES-mediated translation [22], [23], [27], [28], [67], [68]. Differential expression of PTB homologues has been shown to be associated with the neurovirulence of TMEV and PV [19]. In this study human PTB1 was introduced into cells deficient in propagation of the chimeric virus PV1(RIPO), thereby rescuing its growth defect. Specifically PV1(RIPO), if supplied with an excess of hPTB1, can grow in a mouse cell line (L20B). Two different types of functional assays were performed: (i) viral propagation in these cells and (ii) translation and replication activity of a luciferase replicon in these cells.

Numerous studies have shown that the ITAFs directly act on the IRES elements [69], [70]. It is, therefore, reasonable to assume that the expression of hPTB1 in mouse cells stimulates HRV2 IRES activity, either at the stage of assembling the proper IRES structural formation, or perhaps at the stage the subsequent ribosome binding and internal initiation of translation.

The observed phenotypes of PV1(RIPO) in mouse cells and in *CD155* tg mice make the mouse cell lines an excellent system for the isolation of *trans*-acting factors required for the function of the HRV IRES. In the mouse cells, the phenotype of PV1(RIPO) is more severe than in human neuronal cells, which is reflected by absence of viral growth and translational activity even at physiological temperature [20]. There may be several reasons for the differences in the phenotype. The mPTB, although abundantly expressed in L20B cells, does not seem to be capable of activating the HRV2 IRES. In sharp contrast, the expression of hPTB1 in L20B cells increased the activity of HRV2 IRES-mediated translation and, as a result, the growth of PV1(RIPO). A 10-fold increase in IRES activity resulted in more than a 100-fold increase in viral RNA replication (Fig. 5) and about a 100-fold increase in PV1(RIPO) growth in L20B cells (Fig. 3B). However, the expression of the hPTB1 in L20B cells could not completely restore the HRV2 IRES activity such as the IRES-mediated translation (Fig. 5) and the viral growth (Fig. 3B) did not reach titers as high as wt PV in these cells. These results strongly indicate that in addition to hPTB1, HRV2 IRES requires additional factors for efficient internal initiation of translation. Earlier studies by Hunt *et al.* [24], [25] support our results where the authors found that unr and another protein p38 act synergistically with PTB to promote translation dependent on the HRV IRES at highest level.

It was not surprising that only a small number of mutations in the IRES of PV1(RIPO) was required to change the PTB requirement of the mouse cell-adapted PV1(RIPO), R-1235. This observation strongly suggests that the interaction of proteins must have been changed because of i) the changes in the structure of IRES due to the introduction of mutations or ii) the creation of a potential mouse protein binding site (s) by the introduction of mutations. A similar observation was reported in a study with TMEV where introduction of a second site mutation in the viral IRES generated a binding site for nPTB leading to efficient translation initiation and restoration of neurovirulence to wt levels [19].

PTB is an abundant cellular RNA binding protein which is involved in alternative splicing of host cell mRNA [53], [71]. In its natural steady state the protein shuttles between nucleus and cytoplasm [72], with the great majority of protein being localized in the nucleus [73], [74]. During poliovirus infection, however, PTB re-localizes to the cytoplasm [23], [28] where poliovirus has evolved the capacity to hijack the protein for its own purposes to stimulate viral translation. This cytoplasmic accumulation of nuclear protein is not per-chance, but likely a direct consequence of the proteolytic targeting of the nuclear pore complex by picornavirus proteinases [75], [76], [77], [78]. Later in the infectious cycle the accumulated cytoplasmic PTB is degraded by PV 3C^pro^, a process possibly involved in the switch from translation to RNA replication [23].

Our data confirms that PV1(RIPO) was able to make viral proteins only in the cells expressing hPTB1 (Fig. 6B) whereas growth of PV1(M) and R-1235 were independent of hPTB1 in mouse L20B cells (Fig. 6C and 6D respectively). This observation was further supported by our pull-down assay where PV1(RIPO) IRES interacted with hPTB1 only but not with mPTB (Fig. 7). In summary, our findings demonstrated that, although both the PV IRES and the HRV IRES are type I IRES elements, their requirements for PTB are different in mouse cells.

The importance of IRES-dependent tissue and host tropism of picornavirus has led to a significant effort to understand IRES function. Although the structure of the IRES RNA is a primary focus of these studies, the dynamic nature of IRES-ITAF complexes (IRESome) that can be best described as IRES-protein complexes interacting with the small ribosomal subunit, the precursor to the initiation complex, have yet to be solved. The results presented in this study in combination with our previous findings show that the rescue of the PV1(RIPO) growth defect could be achieved in three different ways, either by 1) selection of adaptive mutations, 2) reducing the growth temperature, or 3) modifying the complement of ITAFs in restricted cells. All three mechanisms can conceivably modulate the thermodynamics of RNA folding, by lowering the free energy δG of the active PV1(RIPO) IRES conformation. This in turn may contribute to the formation of the IRESome, thus, facilitating the subsequent interaction with the host cell ribosome and initiation of viral translation. We showed that the available form of PTB in mouse cells (mPTB) is unable to interact with the PV1(RIPO IRES) at 37°C. Therefore we propose that in the first two mechanisms, the mouse cell-adapted mutations or the reduced growth temperature enable the HRV2 IRES to fold properly and in turn to interact with mPTB and/or other unknown factors and these interactions probably recruit the ribosomes to the viral RNA-protein complex. In the third mechanism, hPTB1 supplementation, possibly with the help of other factors, may function as chaperones to enable the HRV2 IRES to form an IRESome and potentially allows the recruitment of ribosomes.

Overall these studies have provided new insights into the interactions of PTB and its mouse homolog with PV1(RIPO) IRES and potential mechanisms of a functional IRESome formation.
